# Long-Term Chironomid Emergence at a Karst Tufa Barrier in Plitvice Lakes National Park, Croatia

**DOI:** 10.3390/insects15010051

**Published:** 2024-01-11

**Authors:** Valentina Dorić, Ivana Pozojević, Viktor Baranov, Zlatko Mihaljević, Marija Ivković

**Affiliations:** 1 Division of Zoology, Department of Biology, Faculty of Science, Rooseveltov trg 6, 10000 Zagreb, Croatia; valentina.doric@biol.pmf.unizg.hr (V.D.); ivana.pozojevic@biol.pmf.unizg.hr (I.P.); zlatko.mihaljevic@biol.pmf.unizg.hr (Z.M.); 2 Doñana Biological Station EBD-CSIC, C/Americo Vespucio, 26, Isla de la Cartuja, 41092 Sevilla, Spain; viktor.baranov@ebd.csic.es

**Keywords:** non-biting midges, diversity, phenology, temperature, organic matter

## Abstract

**Simple Summary:**

Chironomids or non-biting midges, which are a diverse group of insects, can be found in various freshwater habitats. Plitvice Lakes National Park in Croatia, known for its rich freshwater environment, is an ideal location for studying these insects in the long term. Our study aimed to uncover the types of chironomids present, understand their seasonal patterns, and identify factors influencing their emergence in Plitvice Lakes. For 14 years, we set up traps at a tufa barrier within the National Park to collect chironomids on a monthly basis. During this time, we discovered more than 80 chironomid species. Interestingly, we found that water temperature and organic matter were the key factors affecting chironomid emergence in this area. Toward the end of our study, we noticed that the time these insects spent in flight seemed to increase. Although we have not yet found statistical significance, this could be related to the higher water temperatures in winter. In summary, our research sheds light on the fascinating world of chironomids in Plitvice Lakes, highlighting their diversity, seasonal patterns and the environmental factors influencing their behavior.

**Abstract:**

Chironomids are found in all types of freshwater habitats; they are a ubiquitous and highly diverse group of aquatic insects. Plitvice Lakes National Park is the oldest and largest national park in Croatia and consists of numerous and diverse freshwater habitats, making the area an ideal location for long-term research into the chironomid emergence patterns and phenology. The main objectives of this study were to identify the composition of the chironomid community, determine the phenology of the identified species, and assess the main factors influencing their emergence in Plitvice Lakes. During 14 years of research, more than 13,000 chironomids belonging to more than 80 species were recorded. The most abundant species was found to be *Parametriocnemus stylatus*. The highest abundance of chironomids was recorded in lotic habitats with faster water current over substrates of moss and algae and pebbles. Water temperature and the availability of organic matter were found to be the main factors that drive chironomid emergence at the tufa barrier studied. In the last years of this study, a prolonged flight period was observed. Although this is not statistically significant (at this stage of the study), it could be due to a higher water temperature in winter.

## 1. Introduction

The emergence of aquatic insects is the process by which immature insects complete their metamorphosis and emerge from the water [1]. This process creates aquatic–terrestrial linkages through the exchange of energy and matter between the two habitats [2]. The insects that emerge from the water are an important food source for terrestrial predators such as spiders, ground beetles, birds and bats [3,4,5]. Photoperiod (length of day) and water temperature are considered to be the key environmental factors influencing the duration and date of peak emergence [6].

Chironomids are among the most abundant and species-rich aquatic insects. They inhabit all types of freshwater as well as semi-terrestrial, terrestrial, marine and even subterrestrial habitats [7]. Chironomids comprise a large proportion of the benthic fauna and therefore are an important functional component in aquatic ecosystems performing crucial ecosystem functions including aquatic–terrestrial linkages, bioturbation, water remediation and carbon burial [7,8,9]. Due to the close link between the immature stages (larvae and pupa) and the aquatic environment, as well as the great diversity within the group, they are often used as valuable indicators of environmental health [7,10,11]. The larvae are an important food source both in aquaculture and in nature as they are rich in protein, lipids, vitamins and minerals [12]. Most species of adult chironomids have reduced mouthparts and are functionally “aphagous”, meaning that some of them do not feed during the imaginal phase. They also have a very short life span of only one day, which means that their main purpose after emergence is reproduction [13,14]. Although adult chironomids are not hematophagous, they are sometimes considered as pestiferous, creating problems as nuisances and public health risks [12,15].

Plitvice Lakes National Park is a UNESCO World Heritage Site and the oldest and largest national park in Croatia. It is also recognized as one of the biodiversity hotspots [16]. Plitvice Lakes was chosen as a study site because the anthropogenic impact on the system is minimal [17]. Ongoing research on insect emergence in Plitvice Lakes has already provided some valuable information on the phenology and ecology of various aquatic insects: namely, Trichoptera [18], Ephemeroptera [19,20], Plecoptera [21], Odonata [22], Megaloptera [23], Neuroptera [24] and various Diptera families [16,25,26,27,28,29,30,31,32].

The goal of this study was to determine how the emergence phenology of chironomids, as the abundant taxon, may be controlled by environmental factors.

We hypothesized that community dynamics and structural changes in a diverse insect group (here exemplified by chironomids) will reflect the environmental conditions of the ecosystem they inhabit. To test this hypothesis, this study needed to achieve the following specific objectives: (1) identify the composition of the chironomid community and determine the phenology of individual species; (2) assess the environmental factors influencing chironomid emergence; (3) estimate species preferences for specific microhabitat types; and finally (4) determine the magnitude of barrage lake outlet-to-land linkages by emerging chironomids.

## 2. Materials and Methods

### 2.1. Study Area and Study Design

Plitvice Lakes National Park encompasses a network of 16 barrage lakes along with numerous smaller, unnamed lakes interconnected by tufa barriers. Tufa, a result of the precipitation and encrustation of calcium carbonate from the water by aquatic plants and microorganisms under stable water chemistry conditions [33], plays a pivotal role in shaping the park’s landscape. The formation of tufa barriers is a dynamic process influencing lake modifications and water-level fluctuations over time [34]. Although initially perceived as natural lake outlets situated between lakes, research by Šemnički et al. [18] highlights that tufa barriers offer diverse microhabitats akin to stream environments.

This study focuses on the Kozjak–Milanovac tufa barrier, linking the largest lake, Lake Kozjak, to Lake Milanovac. Refer to Dorić et al. [31] for the map of the study area.

**Figure 1:**
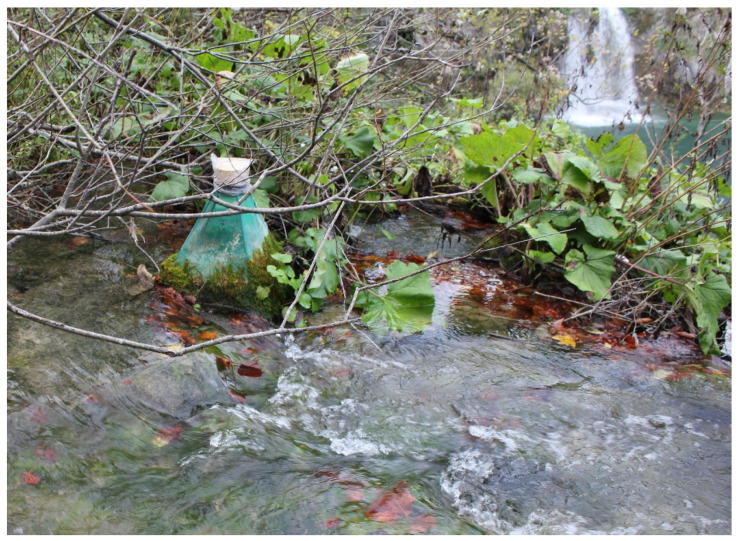
Pyramid-shaped emergence trap at the Kozjak–Milanovac tufa barrier.

To collect adult aquatic insects, emergence traps were deployed from February 2007 to December 2020, covering a span of 14 years. Six pyramid-shaped traps, each with a 45 × 45 cm base and a height of 50 cm, featuring 1 mm mesh nets (Figure 1), were strategically placed in different microhabitats and emptied monthly. The pyramid is slightly elevated from the river bed to allow free movement of the insect larvae. This opening was also cleaned monthly from different vegetation overgrowth in order to preserve microhabitat velocity and composition within the trap. A collecting container, housing a 2% formaldehyde solution with a few drops of detergent, was affixed to each trap. Benthic insect larvae could freely move in and out of the submerged trap base. During monthly field collection trips, adults were preserved in 80% ethanol. Statistical analyses focused on male chironomids identified to species level [35,36,37,38,39,40,41,42], as identifying females to the species level is generally not feasible. As emergence traps are not a selective method, many other groups of aquatic insects were also sampled. These were also processed in many research articles with a comprehensive overview of these findings unified in Ivković et al. [43].

Water temperature, measured twice daily, was recorded using HOBO Pendant Temperature Data loggers (Part UA-001-XX, Onset, Bourne, MA, USA). Average daily discharge values were sourced from the Croatian Meteorological and Hydrological Service. The water levels remained stable thought the study period, meaning that all of the pyramid traps remained submerged to a level between the gathering container and the opening for free larval movement. Monthly measurements of current velocity were conducted using a P-670-M series device (Dostmann electronic, Wertheim, Germany). Simultaneously, conductivity, oxygen saturation, and pH were measured using WTW probes (WTW Oxi 330/SET, WTW pH 330, and WTW LF 330; WTW Wissenschaftlich-Technische Werkstätten GmbH, Weilheim, Germany). Alkalinity was determined monthly through titration with 0.1 M HCl, employing methyl orange as the titration indicator. Chemical oxygen demand (COD) data were provided by the National Park.

### 2.2. Assessment of Factors Influencing Chironomid Phenology in Plitvice Lakes

Canonical Correspondence Analysis was used to quantify the total variation in the chironomid community, while Interactive Forward Selection was used to identify the key environmental parameters influencing chironomid emergence. Bonferroni correction was applied to the *p*-values. These analyses were performed in CANOCO version 5 [44]. Monthly chironomid abundance was tested for correlation with monthly water temperatures, discharge and water velocity using Spearman correlation in IBM SPSS Statistics ver. 27.0 [45].

To investigate the phenological shifts of the species over time, three phenophases for the entire chironomid community were considered:

(1) Month of peak emergence (month of occurrence of the median point of emergence, i.e., when 50% of individuals had emerged);
(2) Duration of emergence (number of months between the start and end of emergence); and
(3) The first appearance (the month in which the species emerged for the first time).

The *mblm* package [46] in R was used to fit a median-based linear model (MBLM) to examine the relationship between each of the selected phenophases and time. The MBLM approach utilizes the median instead of the mean, is robust to outliers, and therefore provides a more reliable estimation of the regression coefficients [46]. The results of the MBLM are reported as MAD (median absolute deviation) and *p* value. The phenophases were tested for correlation with the mean water temperature of each month and the mean annual COD using Spearman’s correlation in IBM SPSS Statistics ver. 27.0 [45].

### 2.3. Microhabitat Preferences

The emergence traps were set at specific microhabitats present at the tufa barrier at that time. Three microhabitats were present: pebbles (1); moss and algae (2); and sand (3). Two emergence traps were set on pebbles, two were set on moss and algae, and two were set on sand. However, in June 2013, it was observed that one of the “pebble traps” became a “moss and algae trap”, and in November 2013, it was observed that one of the “sand traps” became a “pebble trap”. This was considered when carrying out the analyses. When linking the preference for a microhabitat type to specific chironomid species, only those that had a total abundance of more than 190 individuals throughout the study period were used, resulting in 10 species considered for this analysis (almost 50% of the total community). Differences in water velocity, total abundance, species richness and abundance of each of the 10 most abundant species between the three different microhabitat types (1/2, 1/3, 2/3) were tested using the Kruskal–Wallis test and Dunn’s Multiple Comparison test for post hoc pairwise comparisons. This was performed in IBM SPSS Statistics ver. 27.0 [45].

### 2.4. Determination of the Magnitude of Chironomid Emergence

To determine the magnitude of chironomid emergence, chironomid abundance per square meter was calculated. The dry mass was determined only for chironomids emerging from April to August, as emergence was highest during these months and it was possible to collect at least 30 individuals in one month. The average chironomid dry mass was determined by drying at least 30 individuals in each spring/summer month and weighing them using the Mettler Toledo Gold Balance JE503G scale. To estimate the annual emergence magnitude per square meter, the chironomid abundance per square meter per year was multiplied by the average chironomid dry mass of the respective year. The results represent a very rough approximation of annual chironomid emergence.

The average annual dry mass of chironomids was tested for correlation with the following factors: average annual water temperature, average annual discharge and average annual COD using Spearman’s correlation in IBM SPSS Statistics ver. 27.0 [45].

All of the plots were created using the R package *ggplot2* [47]. The R software version 4.2.3 [48] was used to run the two packages mentioned above.

## 3. Results

A total of 13,522 chironomid adults were collected, of which 7797 were males belonging to 81 species. In the whole study of the site, Trichoptera, Plecoptera, Ephemeroptera, Odonata and other Diptera taxa were also abundant and analyzed in detail in Ivković et al. [43]. Most chironomid species belonged to the Orthocladiinae (32 species), and the fewest species (1) belonged to Prodiamesinae. Overall, 20 species of Chironomini, 15 species of Tanytarsini, 11 species of Tanypodinae and 2 species of Diamesinae (Table S1) were recorded at the tufa barrier during the study period.

**Figure 2:**
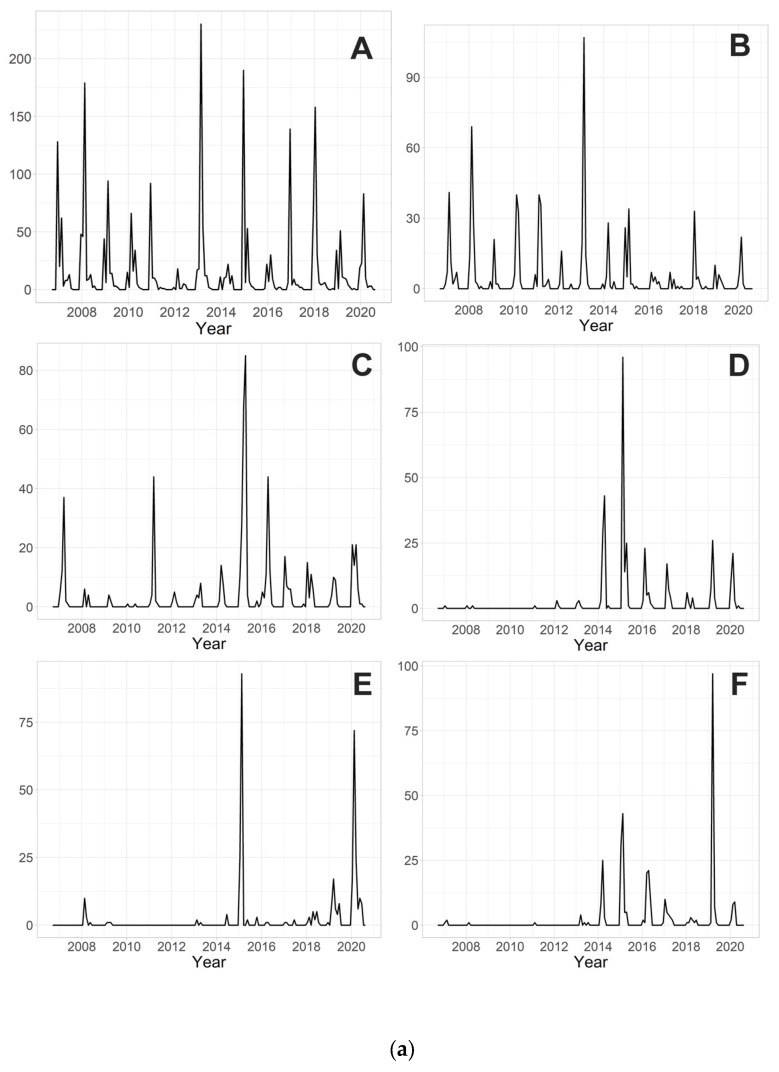

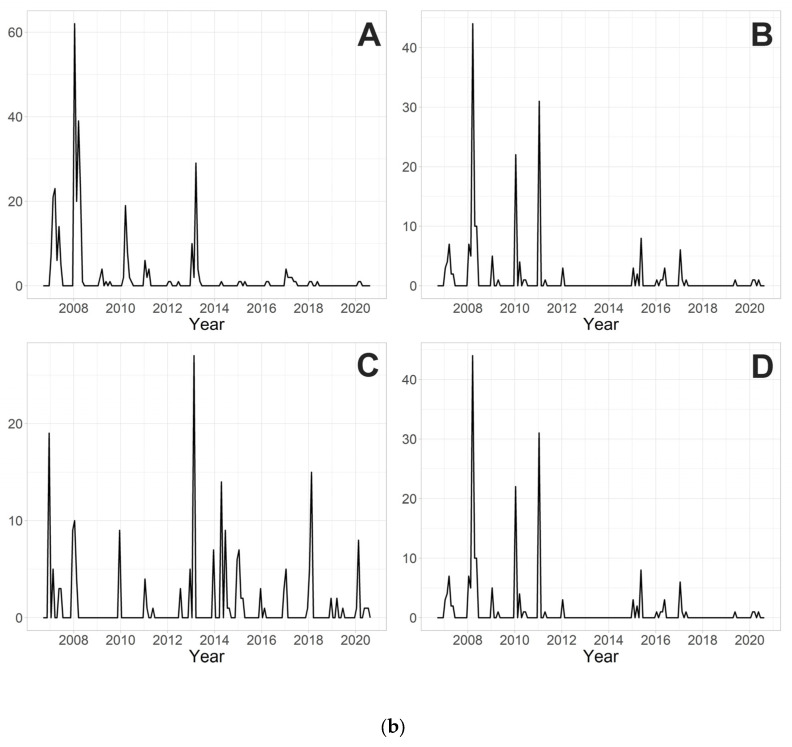
(**a**). Monthly abundances (the number of individuals caught in the traps from a surface area of approximately 0.4 m^2^, i.e., 45 × 45 cm; in a time period of one month) of (**A**) *Parametriocnemus stylatus*; (**B**) *Tanytarsus brundini*; (**C**) *Tanytarsus arduennensis*; (**D**) *Rheopelopia eximia*; (**E**) *Rheotanytarsus curtistylus*; and (**F**) *Rheotanytarsus reissi* during 14 years of research. (**b**). Monthly abundances (the number of individuals caught in the traps from a surface area of approximately 0.4 m^2^, i.e., 45 × 45 cm; in a time period of one month) of (**A**) *Tanytarsus signatus*; (**B**) *Paratrichocladius skirwithensis*; (**C**) *Parametriocnemus* sp. 2; (**D**) *Polypedilum scalaenum* during 14 years of research.

The 10 most abundant species during the 14 years of research are *Parametriocnemus stylatus* (Spaerck, 1923) (2491 individuals), *Tanytarsus brundini* Lindeberg, 1963 (799), *Tanytarsus arduennensis* Goetghebuer, 1922 (614), *Rheopelopia eximia* (Edwards, 1929) (378), *Rheotanytarsus curtistylus* (Goetghebuer, 1921) (351), *Rheotanytarsus reissi* Lehmann, 1970 (345), *Tanytarsus signatus* (van der Wulp, 1859) (344), *Paratrichocladius skirwithensis* (Edwards, 1929) (315), *Parametriocnemus* sp. 2 (202) and *Polypedilum scalaenum* (Schrank, 1803) (194). Their monthly abundances over the years are shown in Figure 2a,b.

The observed seasonal succession of species over the investigated period, 2007–2020, is presented in the Supplementary Material, Table S1. The data were pooled from all traps and years. In general, chironomids were found to emerge throughout the year. The highest species richness was found in June (60), which was followed by May (52) and July (47). The lowest species richness was recorded in January (3), which was followed by December (7), March (8) and February (9).

### 3.1. Assessment of Factors Influencing Chironomid Phenology in Plitvice Lakes

The highest average annual water temperature (12.8 °C) was recorded in 2020 and the lowest (11.03 °C) was recorded in 2010 (Table S2). The highest average annual discharge was measured in 2014 (5.58 m^3^s^−1^) and the lowest was measured in 2011 (1.42 m^3^s^−1^).

The total chironomid abundance was positively correlated with water temperature (r(168) = 0.74; *p* < 0.001) and negatively correlated with discharge (r(168) = −0.23; *p* = 0.003) and water velocity (r(168) = −0.25; *p* = 0.001). For thorough explanations of the changes in the environmental parameters, refer to Dorić et al. [31].

The total variation in the chironomid community was 4.043 (Axis 1 = 0.26, Axis 2 = 0.14), and the explanatory variables tested accounted for 12.7% of the variation. Water temperature (Table 1) was responsible for most of the variation within the chironomid community (5.1%) compared to all other variables tested.

**Figure 3:**
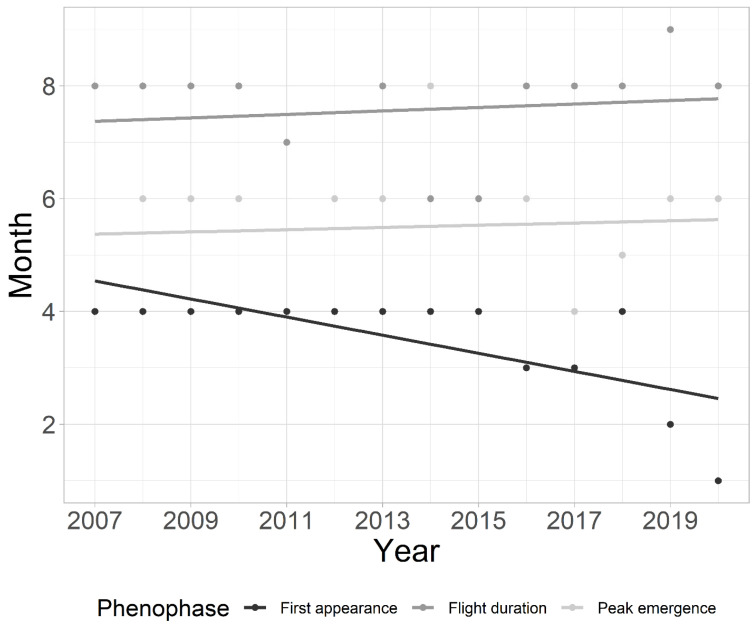
Mean annual shifts in phenology for the entire chironomid community studied over a 14-year period at a tufa barrier in Plitvice Lakes National Park, Croatia.

The earlier first appearance in the whole community was found in the later years of the study (MAD = 0.08; *p* < 0.05), as was the prolonged flight duration (Figure 3). The estimated duration of flight is 0.14 months longer each year (MAD = 0.12; *p* < 0.01). However, the peak of community emergence did not shift within the analyzed period (MAD = 0; *p* > 0.05) (Figure 3). Community flight duration was positively correlated (r (14) = 0.738; *p* < 0.01), while first appearance was negatively correlated (r (14) = −0.633; *p* < 0.05) with the COD. Community flight duration was positively correlated with higher values of mean water temperature in February (r (14) = 0.65; *p* < 0.05) and March (r (14) = 0.55; *p* < 0.05). Peak emergence was negatively correlated with higher water temperature values in May (r (14) = −0.58; *p* < 0.05).

### 3.2. Microhabitat Preferences

**Figure 4:**
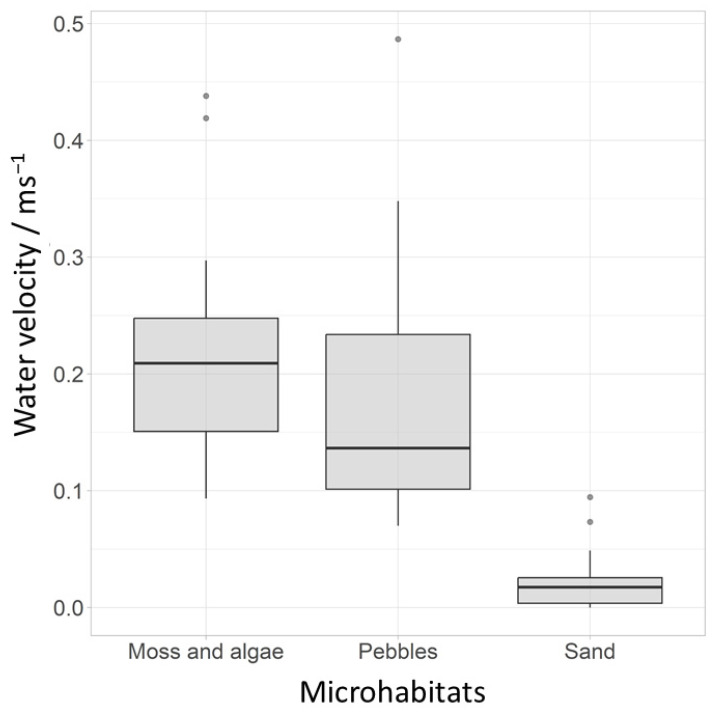
Average water velocity (m s^−1^) at different microhabitats (grey dots represent outliers).

A total of 10 species, accounting for 55% of the total abundance in these 14 years, were used to assess the microhabitats. Most individuals emerged on moss and algae substrate, followed by pebbles, and the fewest individuals emerged in traps attached to sandy substrate. Complete tables can be found in the Supplementary Materials (Table S3). The Kruskal–Wallis test showed significant differences between the tested microhabitats and water velocity and all tested species except *Parametriocnemus* sp. 2 and *T*. *brundini*. The greatest differences in species composition of the chironomid community were observed between sand and the other two microhabitats. The water velocity was lowest over the sand substrate (0.02 m s^−1^) compared to the other two substrates (pebble: 0.17 m s^−1^, moss and algae: 0.21 m s^−1^) (Figure 4). Nine of the species tested preferred moss and algae and pebbles as microhabitats, while two (*Polypedilum scalaenum* and *Tanytarsus signatus*) preferred sand.

### 3.3. Determination of the Magnitude of Chironomid Emergence

**Figure 5:**
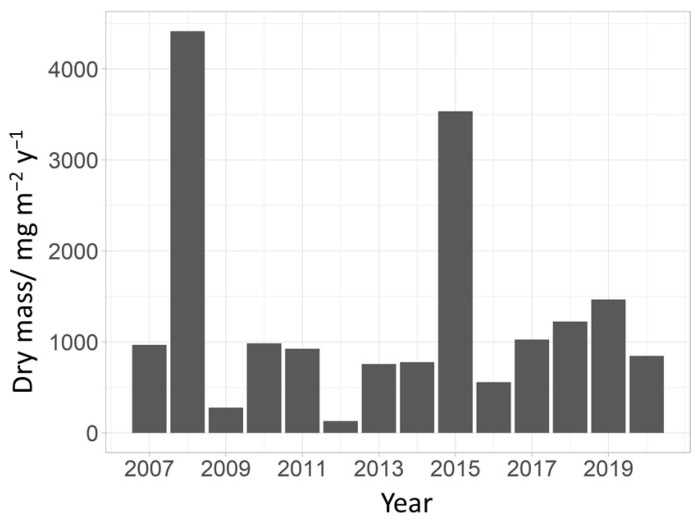
Annual mass of emerged chironomids (mg m^−2^y^−1^) from the six emergence traps at a tufa barrier over the analyzed period. All masses are measured as dry mass.

The total chironomid emergence varied greatly during the study, as did the annual chironomid abundance. The highest production of emerged chironomids from the tufa barrier was recorded in 2008 with 4415.84 mg m^−2^y^−1^. The lowest production was recorded in 2012 with 130.19 mg m^−2^y^−1^ (Figure 5). The average annual dry mass did not correlate with any of the tested parameters (mean annual water temperature, discharge and COD).

## 4. Discussion

### 4.1. Chironomid Community at a Tufa Barrier

In 2011 and 2012, Plitvice Lakes underwent an extreme drought [32], after which the chironomid abundances started to decline. After 2012, the period of extreme discharge generated by prolonged and more intense rainfall set in, and at the same time, the duration of winter snow cover decreased [31]. This combination of events may have led to a drying up of underground water reserves and turned Plitvice Lakes from the former snowfall and groundwater fed system into a more spurious rainfall-dependent system [31]. Discharge levels directly affected other variables such as current velocity, which in turn affected microhabitat conditions and chironomid assemblages therein [31].

These environmental changes have resulted in changes in chironomid phenology. For the most abundant species in this study, *P*. *stylatus*, two or three generations per year are common, as we have recorded. The species usually prefers slow flowing streams and microhabitats that are protected from fast currents. However, this species was found to be the most abundant in the samples with increased discharge and water velocity in Plitvice Lakes [31].

The main food source of *P. stylatus* is slowly decaying organic matter [49]. Although *P. stylatus* was still the dominant taxa in the community, the overall abundance of this species decreased. High discharges, that occurred frequently at the study site, caused the leaves to be washed away, which would otherwise slowly decay and provide *P*. *stylatus* with a preferred microhabitat as well as a food source [50]. The abundances of *Parametriocnemus* sp. 2 fluctuated over the years with the highest abundance recorded in 2013. This species is probably a new species that has yet to be described. It was also found in all types of microhabitats indicating possibly a species that is less dependent on levels of decaying organic matter then other species of the genus [49].

The genus *Tanytarsus* is very heterogeneous, but unfortunately, the ecology of the individual species of the genus is not well known. It is known that the larvae build long, soft tubes and also burrow into the sediments [51]. In our study, the abundances of *T. brundini* and *T. signatus* decreased, while the abundance of *T. arduennensis* increased over the years, possibly suggesting that the latter species is more resilient to the spurious nature of the discharge regimes in the system [31]. The abundances of *Rheopelopia eximia*, *Rheotanytarsus curtistylus* and *R*. *reissi* increased over the studied period. All these species prefer faster flowing streams and *R*. *eximia* can often be found creeping among tubes of other species of the genus *Rheotanytarsus* [14].

We observed the decline in the *P. skirwithensis* abundance, as shown in a monthly abundance graph. The species primarily inhabits spring and groundwater-dependent ecosystems [52], which means that the altered discharge regimes observed at the study site may have had an unfavorable impact on the species’ phenology.

*P*. *scalaenum* is also a species for which a decline in abundance was observed. The larvae of this species inhabit sandy soils [53]. Unfortunately, the extremely high discharges recorded in Plitvice Lakes, which led to the flushing of the sand have also caused the loss of the preferred habitat for *P*. *scalaenum*.

### 4.2. Factors Influencing Chironomid Phenology in Plitvice Lakes

The water temperature is the factor that best explains chironomid community composition as previously demonstrated [6,54,55]. The rate of chironomid larval development increases with the water temperature [14]. The onset of chironomid emergence in temperate areas is associated with the rising spring temperatures [7]. However, temperature, and the other environmental variables tested, explained a small percentage of the total emergence timing variation of the chironomid community. This suggests that inter and intra-species relationships may have a stronger importance in shaping the chironomid phenology [7].

Predation on larvae and competition for food influence the timing of emergence [7]. The highest abundance of chironomids was observed in the spring and summer months when the water temperature was higher. We can therefore confirm that water temperature is an important factor triggering chironomid emergence at the tufa barrier. A negative correlation between discharge and chironomid abundance is most likely due to the fact that higher discharge occurs during the colder, rainy period of the year (late autumn, winter and early spring) when chironomid abundance is lower. On the other hand, extremely high discharge during warmer periods of the year may result in lower chironomid emergence due to substrate disturbance and drift [27,54,56].

The chironomid flight duration Increased, and they started to emerge earlier during the study period. When the water temperature was higher in February and March and there was more organic matter in the system, chironomid emergence started earlier and lasted longer. The peak time of emergence varied over the years, but it was mostly in June. We did not detect significant shifts in peak emergence, which was most probably due to the monthly collection efforts, where daily or weekly changes can be overlooked [57]. In the period analyzed, the peak emergence occurred twice in May (2017 and 2018) and twice in July (2014 and 2019). The earlier peaks of emergence in May (in 2017 and 2018) could have been a result of the higher water temperature in the preceding months and the above average water temperature in May. The later peaks in July (in 2014 and 2019) could be the result of below average water temperatures that occurred in May of these years (12.4 °C in 2014 and 11.5 °C in 2019). In comparison, the average water temperature measured in May was almost 14 °C. Overall, peak emergence has not shifted significantly over the years, but during the last 4 years of research, a peak was detected only once in the “usual” peak abundance month, June of 2020. If this shift in peak abundance timing continues, it could lead to potential problems for terrestrial communities. Changes in the phenologies of aquatic and terrestrial insect communities could lead to asynchrony between the demand of insectivorous consumers and what is available in the environment [5].

### 4.3. Microhabitat Preferences

The emergence traps attached to the sand substrate were characterized by the lowest water velocity, but during 2013, the sand was washed away from the more exposed of the two traps and the pebbles were left behind. This was probably a result of the unusually high discharge and high water velocity in October 2013, which was facilitated by the unusually low water level of 2011/2012. This is yet another insight into the dynamics of the everchanging microhabitat mosaic that highlights the importance of microhabitat selection and monitoring in this type of long-term research in order to understand the dynamics of our target group of organisms. For instance, an emergence trap, which still stands on sandy substrate today, is in a very sheltered location amidst emergent macrophytes (*Cladium mariscus*) and shows distinctly different chironomid assemblages in comparison to other traps throughout the research period. Also, the moss that was observed in one of the pebble traps in the emergence trap P3 in June 2013 has most likely spread from the neighboring habitat into the emergence trap, which is most probably the cause of increased abundances of chironomid individuals in this trap in the second part of the research.

Most of the species used for the microhabitat assessment did not show a clear association to only one microhabitat type, with the exception of *P*. *scalaenum* and *T*. *signatus*, which showed a clear preference for sandy substrate and whose occurrence at the tufa barrier has decreased over the years due to the flushing of this substrate. Most of the other species seem to avoid slower flowing water and sandy substrate, which is probably due to its homogeneity. Sand does not provide a variety of microhabitats [58], and slower water velocity could lead to the deposition of organic matter and its slow decomposition [59,60].

### 4.4. Magnitude of Chironomid Emergence

The chironomid emergence production rate of 130 to 4400 mg m^−2^ y^−1^ corresponds to an oligotrophic to mesotrophic production of the system. However, the trophic status of the system is a rough estimate based only on a part of the fauna present in it, so it should be taken accordingly. The highest production rates correspond to years of highest chironomid abundances, and the same goes for the years with lowest rates. Lake Kozjak is known to normally fluctuate between these two trophic states [43,61]. This study shows that in years of high chironomid emergence, up to 4400 mg m^−2^ y^−1^ of chironomid biomass is available to terrestrial animals either directly as prey or indirectly as carcasses that decay on the soil surface [62]. When these chironomids die, their bodies decompose and add nutrients to the soil, mainly resulting in nitrogen and phosphate enrichment [4].

## 5. Conclusions

Changes in the composition of the chironomid community could affect its availability as a food source for other organisms, but they could also lead to changes in the bioturbation rate. Chironomid emergence was strongly influenced by water temperature and the amount of organic matter available in the system. Chironomids at a tufa barrier now have a longer flight duration due to higher water temperatures in winter. The ramifications of climate-driven changes include increased water temperature, which could affect peak emergence and thus decouple it from the phenology of insectivorous consumers. This is still not the case for Plitvice Lakes, but on the other hand, most studies that have found shifts in peak emergence have found shifts in days, so our monthly efforts may not be sufficient to detect these shifts. Weekly or even daily collections could lead to a more accurate detection of the shift in peak emergence. Of all species, only two showed a clear preference for sandy substrate, and their abundances are severely declining due to an increased flushing of sediments by high discharge. As chironomid larvae contribute to the recycling of organic matter and form an important trophic link between primary producers and fish, a more in-depth study of chironomid production in conjunction with their deposition on land could be a good way to continue and advance this work.

## Figures and Tables

**Table 1:** The variability of the chironomid community in relation to the environmental variables selected by the Canonical Correspondence Analysis using Interactive Forward Selection.

Environmental Variable	Explains %	Contribution %	Pseudo-F	*p*	P (adj)
Temperature/°C	5.1	24.2	6.5	0.002	0.024
COD_KMnO_4_/mg O_2_ L^−1^	4	19	5.3	0.002	0.024
Oxygen/mg O_2_ L^−1^	1.9	9.3	2.6	0.002	0.024
Nitrites/mg N L^−1^	1.7	8.3	2.4	0.002	0.024

## Data Availability

Data supporting reported results can be provided upon contacting the corresponding author.

## Supplementary Materials

The following supporting information can be downloaded at https://www.mdpi.com/article/10.3390/insects15010051/s1, Table S1: The community of chironomids and their flight periods at tufa barrier Kozjak–Milanovac over the 14-year period (2007–2020); Table S2: Average monthly and annual water temperatures during the study period (2007–2020); Table S3: Microhabitat preferences of the 10 most abundant species at tufa barrier Kozjak–Milanovac.
